# SEX-RELATED DIFFERENCES IN PHYSICAL ACTIVITY PATTERNS OF OLDER INDIVIDUALS LIVING IN THE SARDINIAN BLUE ZONE

**DOI:** 10.1093/geroni/igae098.0232

**Published:** 2024-12-31

**Authors:** Massimiliano Pau, Benedetta Brandas, Maria Chiara Fastame

**Affiliations:** University of Cagliari, Cagliari, Sardegna, Italy; University of Cagliari, Cagliari, Sardegna, Italy; University of Cagliari, Cagliari, Sardegna, Italy

## Abstract

Previous studies demonstrated that individuals born and resident in the Blue Zones (i.e, areas of exceptional longevity) exhibit reduced levels of depressive symptoms, better life satisfaction levels, and adopt more effective coping strategies to deal negative life events. However, few data is available regarding their physical activity (PA) profiles and, in particular, it is not known whether they are sex-related. Based on these considerations, in the present study we explored the effect of sex on a set of metrics which describe amount and intensity of physical activity (PA) in older people living in the Blue Zone of Sardinia (Italy). To this aim, 109 community-dwelling participants (Mage = 81.7 years old, SD = 8.1 years) were monitored for 7 days using a clinically validated wearable accelerometer which provided raw body accelerations as well as percentage of time spent in activities of different intensity namely sedentary behavior (SB), light PA (LPA) and moderate-to-vigorous PA (MVPA). The results showed that women exhibit a better PA profile with respect to men, characterized by significantly higher time spent in MVPA (+37%, p =.008) and reduced SB (-10%, p =.001). Also, those living in the Blue Zone exhibit higher PA levels with respect to peers resident in urban areas of Sardinia, thus suggesting that they adopt a more active lifestyle, which is thought to play a pivotal role in promoting functional and cognitive health in late adulthood. At the same time, futher studies are needed to clarify the nature of sex-related differences here observed.

